# Nutraceutical potentialities of Tunisian Argan oil based on its physicochemical properties and fatty acid content as assessed through Bayesian network analyses

**DOI:** 10.1186/s12944-018-0782-9

**Published:** 2018-06-15

**Authors:** Mohsen Hanana, Hajer Mezghenni, Rayda Ben Ayed, Ali Ben Dhiab, Slim Jarradi, Bassem Jamoussi, Lamia Hamrouni

**Affiliations:** 1 Extremophile Plants Laboratory, Centre of Biotechnology of Borj-Cedria, BP 901, 2050 Hammam-Lif, Tunisia; 2 Laboratory of Forestry Resources Management and Valorization, National Research Institute of Rural Engineering, Water and Forests, P.B. 10, 2080 Ariana, Tunisia; 3 Laboratory of Molecular and Cellular Screening Processes, Genomics and Bioinformatics Group, Centre of Biotechnology of Sfax, PB ‘1177’, 3018 Sfax, Tunisia; 4 Olive Tree Institute, Sousse, Tunisia; 5 General Direction of Forests, Ministry of Agriculture, Hydraulic Resources and Fishing, 30 Avenue Alain Savary, 1002 Tunis, Tunisia; 6 Laboratory of Analytical and Organic Chemistry, High Institute of Continue Education and Formation, 43 Rue de la Liberté, 2019 Le Bardo, Tunisie

**Keywords:** *Argania spinosa*, Carotenoids, Cosmeceuticals, Fatty acids, Nutraceuticals, Polyphenols

## Abstract

**Background:**

Argan oil is traditionally produced by cold pressing in South-western Morocco where rural population uses it as edible oil as well as for its therapeutic properties which give them in counterpart valuable income. Given the economical interest of this oil, several attempts of fraudulency have been registered in the world global market leading to loss of authenticity. Our purpose is to launch a program of Tunisian Argan oil valorization since trees from this species have been introduced sixty years ago in Tunisia. The first step was thus to characterize the physicochemical properties and determine the chemical composition of Tunisian Argan oil in order to assess its quality.

**Methods:**

Physicochemical parameters of oil quality were determined according to the international standard protocols. Fatty acid content analysis of Argan oils was performed by gas chromatography coupled to mass spectrophotometry. A comparative study was realized among Tunisian, Moroccan and Algerian samples differing also by their extraction procedure. The impact of geographical localisation on the fatty acids composition was studied by statistical and modeling Bayesian analyses.

**Results:**

Physicochemical parameters analysis showed interestingly that Tunisian Argan oil could be classified as extra virgin oil. Argan oil is mainly composed by unsaturated fatty acids (80%), mainly oleic and linoleic acid (linoleic acid was positively influenced by the geographical localization (*r* = 0.899, *p* = 0.038) and the P/S index (*r* = 0.987, *p* = 0.002)) followed by saturated fatty acids (20%) with other beneficial compounds from the unsaponifiable fraction like polyphenols and carotenoids. Together with fatty acid content, these minor components are likely to be responsible for its nutraceutical properties and beneficial effects.

**Conclusion:**

Tunisian Argan oil displayed valuable qualitative parameters proving its competitiveness in comparison with Moroccan and Algerian oils, and could be therefore considered as extra virgin edible oil for nutraceutical purposes as well as for cosmetic use.

## Background

Argan tree*, Argania spinosa* (L.) Skeels*,* also known as “iron tree” for its hardy trunk, is an evergreen tree that belongs to the Sapotaceae family and can reach a height of 8 to 10 m [1]. It is endemic of south-western Morocco where it grows over about 828,000 ha [2] but also grows naturally (70,000 ha) in south-western Algeria in Tindouf region [3]. Able to live over 200 years and being well adapted to arid climate, Argan tree would play an important ecological role in protecting against sandy lands progression and wind erosion [4]. In addition, it has multiple purposes, particularly virgin oil production from its fruits seeds which produces economical benefits for rural population. Indeed, each part of the tree can be used in wood manufacturing and by-products (trunk and stems), as fodder (leaves and pulp), oil production (kernels), and constitutes thus a valuable income source [5]. The most important product of the tree is an edible oil extracted from the fruit kernels by diverse extraction processes (traditional, industrial, by press or organic solvent). It has an excellent food quality and a high marketable value, but is also used in traditional medicine, pharmacology and more recently in cosmetology [2].

However, in its natural habitat, Argan tree faces several threatens such as anthropic pressure, overgrazing, weakness of natural regeneration due to seeds over-exploitation, leading to the regression of its growth area. In Tunisia, in the early sixties, a national program of reforestation including Argan tree has been launched. Despite its importance, Argan tree has not been well valorized and was somehow neglected in Tunisia. In this context, our work aims to valorize Tunisian Argan oil, in comparison with the Moroccan and Algerian oils, and to highlight its beneficial nutritional, nutraceutical and cosmetological effects according its physicochemical properties and fatty acid content.

## Methods

### Plant material

**Fig. 1:**
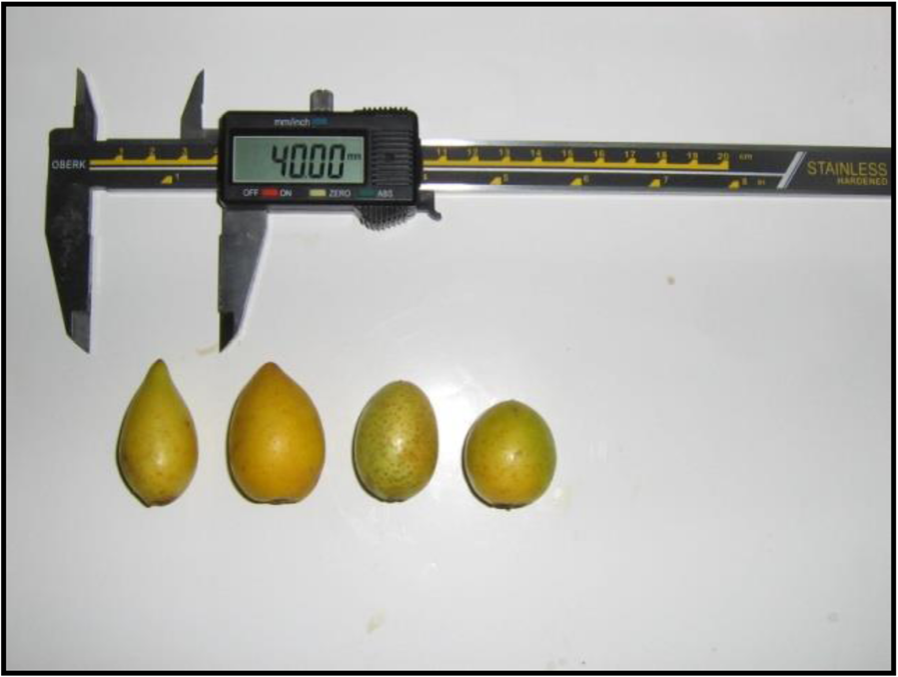
Diverse forms of Argan fruits

Argan fruits were collected from the Botanical Garden of ‘Institut National de Recherches en Génie Rural, Eaux et Forêts’ (INRGREF) at maturity stage (June–July). After harvest, fruits, nut-sized, round, ovoid or conical and yellowish turning color (Fig. 1), were dried into the open air and then stored at 4 °C until their use for oil extraction.

### Methodology

Tunisian samples were submitted to chemical extraction by organic solvent in order to get better yield. Moroccan Argan oil (commercialized as cosmetic oil) was produced by cooperative and prepared by cold pressing extraction. Chemical composition, organoleptic properties and physico-chemical parameters analyses were proceeded on those Argan oil samples.

### Extraction process

#### Sample preparation

**Fig. 2:**
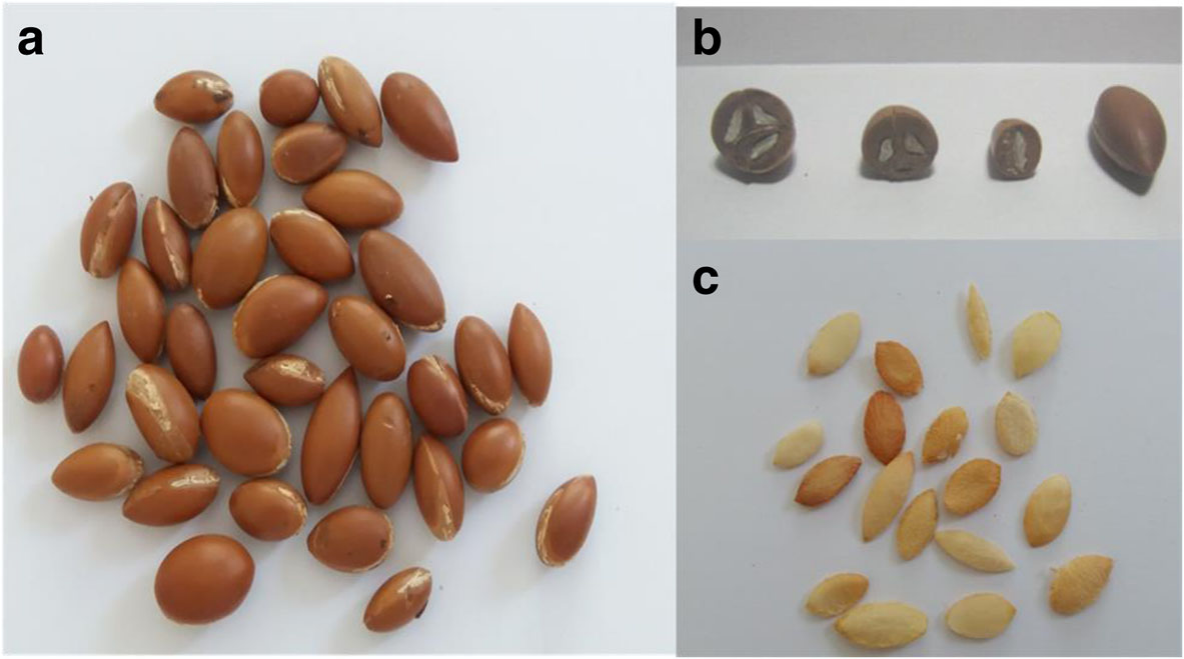
Nuts and kernels of Argan. a: nuts, b: radial cut of nuts showing one, two or three kernels, c: kernels

The first step was to pulp mechanically fruits (40 kg) using scalpel blade to separate the pulp from the nut. The latter was grinded with a mortal to put out the white kernel (Fig. 2).

#### Oil extraction

Kernels were heated at 40 °C during 12 h in oven. Overheating should be avoided to preserve sensory taste from degradation. Oil sample from Morroco was obtained without application of heat treatment nor roasting step since it is intended for cosmetic use. After their dessication, kernels were ground by a grinder to afford brownish dough. Solvent (hexane) extraction in a Soxhlet type apparatus was used for Tunisian Argan sample while cold pressing procedure was applied to Moroccan one. Time of extraction in the Soxhlet was set to 6 h, and then hexane was evaporated and removed from the extracted oil in using a rotary evaporator with an operating bath temperature at 60 °C to yield a solvent vapor temperature of 40 °C which is subsequently condensed at 20 °C.

### Argan oil analysis

#### Physicochemical quality parameters characterization

##### Yield determination

Yields were determined in terms of nut/fruit, kernel/nut and oil/ kernel.

##### Relative density

Relative density (g/ml) of Argan kernel oil was determined at 20 °C.

##### Refractive index

The determination of Argan oil refractive index was performed using an Abbé refractometer at 20 °C. According to Ourrach et al. [6], the refractive index parameter would help in detecting the adulteration of argan oil with edible vegetable oils.

##### Free Acidity and Acid Index determination

Free acidity, given as % of oleic acid, and corresponding to the free fatty acids (FFA) present in the oil, was determined by titration of a solution of Argan oil (5 g) dissolved in an ethanolic neutralized solution (30 ml). This solution was then titrated with the NaOH solution (0,177 N) until the phenolphthalein indicator turned slightly pink, indicating the equivalence point had been met. The calculation is determined by the following formula:

$$ \mathbf{Free}\ \mathbf{Acidity}\ \left(\%\right)=\left(\mathbf{V}\ \mathbf{x}\ \mathbf{C}\ \mathbf{x}\ \mathbf{M}\right)/\left(\mathbf{10}\ \mathbf{x}\ \mathbf{m}\right) $$

Where:

- V is the volume (ml) of NaOH solution used for the titration.
- C is the concentration of NaOH solution (mol/L).
- m is the weight of the oil sample.
- M is the oleic acid molecular weight (g/mole).

Acidity Index, corresponding to the necessary amount (mg) of potassium hydroxide to neutralize the free acids present in 1 g of the oil, was determined by titration of a solution of oil (1 g) dissolved in an ethanolic neutralized solution (5 ml) with 0.1 M potassium hydroxide and phenolphthalein indicator as for free acidity determination. Acidity index is thus given by the following formula:

$$ \mathbf{IA}=\left(\mathbf{V}\ \mathbf{x}\ \mathbf{56},\mathbf{1}\ \mathbf{x}\ \mathbf{N}\right)/\mathbf{m} $$

where:

- V is the volume (ml) of KOH solution used for the titration.
- 56,1 is the KOH molecular weight (g/mol).
- m is the weight (g) of the oil sample.
- N is the normality of KOH solution (0,1 N).

##### Total polyphenols content determination

The total polyphenols content was evaluated using the Folin−Ciocalteu colorimetric method.

A triple extraction was proceeded on a solution of Argan oil in hexane and methanol followed by addition of Folin-Ciocalteau reagent to the mixture and the absorption of the solution was measured 1 h later at 725 nm. Total polyphenols content is expressed as “milligram equivalent of Gallic (or Caffeic) acid per kilogram of oil” (mg/kg) or “micrograms of phenols per mg of oil”.

#### Experimental protocol

10 g of Argan oil was dissolved in 25 ml of hexane and extracted three times with 60% aqueous methanol. The methanolic extract was then made up to 50 ml with water and left to stand at least 6 h. To 5 ml aliquot of the methanolic extract add 1,25 ml Folin-Ciocalteau reagent and 15 ml H_2_O_2_. Shake the mixture well and let to stand for 5 min. 2,5 ml of NaOH (6%) and water (sufficient amount to 25 ml) were then added and let to stabilize for one hour. Finally, the absorption was read at 725 nm using uv-visible spectrophotometer. In the same manner, calibration curve is prepared by several dilutions of the aqueous gallic acid solution free from Argan oil. The total polyphenols content is given by the following formula

$$ \mathbf{Total}\ \mathbf{polyphenols}=\mathbf{403},\mathbf{68}\ \mathbf{x}\ \mathbf{A}\mathbf{725}\left(\mathbf{ppm}\right)\kern3em \mathrm{for}\ 10\ \mathrm{g}\ \mathrm{of}\ \mathrm{Argan}\ \mathrm{oil} $$

where:

- A_725_ is the absorbance at 725 nm.

##### Chlorophyll content determination

Chlorophyll content (ppm or mg/kg oil) was determined using a spectrophotometric method as described by Wolff [7].

Absorbance of Argan oil was measured at three wavelengths 630, 670 and 710 nm. The chlorophyll content is given according to the following formula:

$$ \mathrm{Chlorophyll}\ \mathrm{content}=\frac{\mathrm{A}670\hbox{-} \left(\mathrm{A}630+\mathrm{A}710\right)/2}{0,1086\ \mathrm{x}\ \mathrm{L}} $$

where:

- A: Absorbance at the indicated wavelength.
- L: spectrophotometer cell thickness = 1 cm.

##### Carotenoid content determination

Carotenoid content (ppm or mg/kg oil) was determined at 470 nm in cyclohexane (7,5 ml Argan oil in cyclohexane, sufficient amount to 25 ml) using the specific extinction values, by the method of Minguez-Mosquera et al. [8].

$$ \mathbf{Carotenoid}\ \mathbf{content}=\left({\mathbf{A}}_{\mathbf{470}}\mathbf{x}\ \mathbf{25}\ \mathbf{x}\ \mathbf{10000}\right)/\left(\mathbf{2000}\ \mathbf{x}\ \mathbf{7},\mathbf{5}\right) $$

where:

- A: Absorbance at the indicated wavelength.

### Fatty acid composition analysis

The fatty acid composition determination was performed according to the recommendations and the analytical method described in the European Union Commission Regulation 2568/91 [9]. Before starting the CPG analysis, fatty acids have to be converted in methyl esters in order to reduce their polarity, make them more volatile and therefore more detectable.

#### Fatty acid methyl esters (FAME) preparation

The protocol used for the preparation of the FAME is a cold trans-esterification method using a methanolic solution of potassium hydroxide. The methyl esters were prepared by vigorously shaking a solution of Argan oil in hexane (0.1 g in 2 ml) with 0.2 ml methanolic potassium hydroxide solution (2 N). The converted FAME are ready for gas chromatography analysis.

#### GC/MS analysis of FAME

The analysis of the Argan oil composition was carried out by gas chromatography coupled to mass spectrometry (GC/MS) type GC-2014 Shimazu Gas Chromatograph and mass spectrometer, equipped with a flame ionization detector using fused silica capillary column, low polar DB5 capillary column (30 m, internal diameter of 25 mm and a film thickness of 0.25 μm) to obtain individual peaks of the FAME. The inert carrier gas was hydrogen with a total flux of 54 ml/min, a flow rate of 1 ml/min and a pressure of 54.3 KPa. Other parameters were: injector port: 260 °C, detector: 280 °C, split ratio: 1/50. The temperature of the column was maintained for 2 min at 150 °C and increases at a rate of 5 °C/min at 200 °C for 35 min. The total elution time was 47 min. Diluted samples in hexane (1/40 for Tunisian Argan oil and 1/20 for Moroccan samples) of 1 μl were injected manually in the splitless mode. The peaks of FAME were identified by comparing their retention times with those of olive oil (used as a standard). The percentage of a fatty acid is calculated as follows: the area of ​​the peak of the fatty acid considered on the sum of the areas of all the peaks of a chromatogram corresponding to the fatty acids of the mixture.

### Statistical analysis

#### Correlation analysis

Pearson’s correlation analysis was used to test associations between variables. All analyses were performed using R program. Two-sided *P*-values< 0.05 were considered statistically significant.

#### Bayesian network Modelling

To establish relationships between cultivation region, extraction procedure and fatty acids composition in Argan oil, the developed model, based on Bayesian network techniques, was used to learn emerging models in data. Bayesian networks are DAGs composed by nodes (variables of the problem, in this work, we used ten variables: Region, Extraction procedure, Palmitic acid, Stearic acid, Oleic acid, Linoleic acid, Total SFA, Total UFA, P/S index and UFA/SFA ratio) and arcs that encode conditional probabilistic independencies between the nodes. This graphical model is attractive for its aptitude to explain probabilistic interactions connecting variables. In fact, it has proven to capture causal relationships between variables and it can show excellent forecast accuracy even with relatively small sample data sizes. To achieve the mentioned objectives, Bayesian network modelling was used. Our proposed methodology includes the following different stages for building model: data normalization and construction of Bayesian networks. Data normalization consists of a sample data from experimental data. For construction of Bayesian networks, two nodes i and j having a partial correlation are connected by a non oriented edge. The orientation is determined by a heuristic method based on the following test [10]: If B_ij_ = w_jj_ σ_ii_/w_ii_ σ_jj_ > 1, the arc is then oriented from _i_ to _j_ and if B_ij_ = w_jj_ σ_ii_/w_ii_ σ_jj_ < 1, the arc is then oriented from _j_ to _i_. The other edges with B_ij_ = w_jj_ σ_ii_ /w_ii_ σ_jj_ = 1 remained undirected. The graph with all directed arcs constituted the Bayesian network. The advantage of Bayesian network is to deduct all parent nodes which are directly dependent on child nodes. R program was used to analyze obtained data and to draw the Directed Acyclic Graph (DAG).

## Results

### Extraction and yield obtention

Yield calculation showed that yields of nut/fruit and kernel/nut were respectively 25 and 10% for Tunisian Argan. Tunisian Argan oil/kernel yield reached 61,3% similarly to Moroccan (58%) and Algerian (Mostaganem, 66,5%) oils that were also extracted by hexane solvent (Table 1). However, yields of Argan oil, mechanically or handly pressed, were much lower, showing the influence of extraction procedure on oil yield.

**Table 1:** Yields determination according to the origin and procedure of extraction of Argan oil

Sample	Tunisian	Moroccan	Moroccan	Moroccan	Algerian	Algerian
nut/fruit	25	na	27	na	na	na
kernel/nut	10	na	10,8	na	na	na
kernel/fruit	2,5	na	3	2,7	na	na
oil/kernel	61,3	58	30–50	20	55,9	66,5
Protocol of extraction	solvent	solvent [33]	mechanical pressing [34]	hand pressing [35]	solvent [36] (Tindouf)	solvent [36] (Mostaganem)

*na* not available

### Physicochemical properties of Argan oil

Among analyzed and compared oils, Tunisian Argan oil displayed the lowest relative density (0,84), which makes it classified as oleic type according to Karleskind [11].

Approximately, the relative density values of the various Argan oils, wherever they come from (Tunisia, Morocco or Algeria) and regardless the extraction process (solvent extraction, or cold pressure extraction), are found in the 0,84–0,92 range (Table 2) which is below the international standard (UICPA 2.101) range [12]. The determination of the refractive index would help in controlling the authenticity and purity of Argan oil. Similarly to the olive oils [12], the refractive index of all Argan oil samples is around 1,47 attesting to their low level of trienes. Having a refractive index higher than the one of epidermis (1,44) [13], Argan oil would be suitable for cosmetic use to prevent solar radiation damage. Moreover, Tunisian Argan oil displays low values of acidity index (1,85) and free acidity (0,9) that would confer a better oxidative stability. According to the CODEX STAN 33–1981 (ISO 660: 1996) standard [12], Tunisian Argan oil could be considered as extra virgin oil. Origine of Argan samples may be a factor influencing the free acidity, since Moroccan and Algerian free acidity values differ and are respectively considered as virgin and refined, reflecting thus geographical specificity and/or mode of extraction.

**Table 2:** Physicochemical properties of the Argan oil according to the origin and procedure of extraction

Properties	Tunisia solvent extraction	Morocco cold pressing extraction	Morocco Solvent extraction [37]	Algeria solvent extraction [36] (Tindouf)	Algeria cold pressing extraction [38] (Tindouf)	Algeria solvent extraction [36] (Mostaganem)
Density (20 °C)	0,841	0,890	0,906	0,912	0,914	0,918
Refractive index (20 °C)	1470	1473	1468	1465	na	1469
Acidity index	1851	2805	na	na	na	na
Free Acidity (% oleic acid)	0,90	1,05	1,3	0,12	0,8	0,11
Oil quality	extra virgin	virgin	virgin	refined	extra virgin	refined

*na* not available

### Unsaponifiable fraction characterization

Despite its low content (~ 1%), unsaponifiable fraction still represents an important matter, comprising compounds like polyphenols, tocopherols, carotenoids and xanthophylls [14] which also contribute to the Argan oil nutritional value and health properties [15].

The values of polyphenols content in Argan oil vary according to the origin and extraction process, ranging from 10 to 120 ppm, Tunisian Argan oil having an intermediate value of 55 ppm. Given that Argan trees from Algeria were grown under 4–8 millimhos/cm of soil salinity, the high value obtained by the Algerian oil (120 ppm) could be explain by the salinity which is well known to induce polyphenols synthesis within salt stressed tissues as an adaptation response. Even displaying better polyphenols content, the quality of Algerian oil would have been depreciated by salt stress effect. Indeed, Table 3 shows that the Algerian Argan oil [16] has one of the worst and poorest fatty acid composition among analyzed oils, mainly because of negative salinity impact. In all cases, high polyphenols content is associated with antioxidant properties and contributes to oil’s shelf life preservation by preventing oxidative deterioration. Moreover, due to their antiradical activity, polyphenols would protect Argan oil from degradation during storage and cooking [17].

**Table 3:** Polyphenol, chlorophyll and carotenoid contents (ppm) of the Argan oil according to the origin and procedure of extraction

	Tunisian	Moroccan [34]	Algerian [38]
Polyphenol	55	10	120
Chlorophyll	0,009	1,8	1,3
Carotenoid	1,8	0,86	0,74
Extraction procedure	solvent	mechanical pressing	cold hand pressing

While Moroccan and Algerian Argan oils display respectively 1,8 and 1,3 ppm chlorophyll content (Table 3), the one of Tunisia is practically null, showing no contamination by foliar extract. Indeed, traces of chlorophyll in the Argan oil is synonym of contamination by leaves or of fraudulency, and high amount of chlorophyll would lead to detrimental impact on oil quality.

Carotenoid content ranged from 0,74 to 1,8 ppm, Tunisian Argan oil having the highest value. Carotenoid richness of Argan oil warrants antioxidant activity and therefore better protection against free radical damage, higher nutritional value and longer preservation of oil.

### Fatty acids composition of Argan oils

GC coupled to MS allowed us to identify the fatty acid composition of analyzed Argan oils. Fatty acid profiles of Tunisian and Moroccan Argan oils are represented in Table 4 where other samples from Algeria and Morocco differing in extraction procedure have been also mentioned. For all analyzed oils, unsaturated fatty acids (UFA) are predominant, oleic and linoleic acids were the major compounds, making them oleic-linoleic of type. The main UFA was oleic acid, particularly for the Tunisian Argan oil where it reached 52,53%, almost the range of olive oil. However, the Tunisian Argan oil was characterized by the lowest content of linoleic acid (24%), although still being considered as much high than olive oil (7%) [14]. The third and fourth main fatty acids found in Argan oil are palmitic acid (12,3–16,2%) and stearic acid (4,7–7,1%) for whom highest values were registered for Tunisian Argan oil, increasing its grade and qualitative value. The highest P/S index (Polyunsaturated/Saturated index) was found for Algerian Argan oil from Mostaganem (2,16) and the lowest P/S index was found for Tunisian sample (1,04).

**Table 4:** Fatty acids composition of Argan oil according to the origin and procedure of extraction

	Fatty acids	Tunisia solvent extraction	Morocco cold pressing extraction (cosmetic)	Morocco solvent extraction [33]	Morocco cold pressing extraction (edible) [35]	Algeria solvent extraction [36] (Tindouf)	Algeria cold pressing extraction [45] (Tindouf)	Algeria solvent extraction [36] (Mostaganem)
SFA	Palmitic acid C16:0	16,13	14,43	12,79	13,5	13, 84	13	12, 28
	Stearic acid C18:0	7,10	6,17	5,35	5,5	5, 68	5,2	4, 72
UFA	Oleic acid C18:1	52,53	47,17	45,53	47	50,30	44,3	45,02
	Linoleic acid C18:2	24,24	32,22	34,64	33	28, 99	35,8	36, 80
Total SFA		23,23	20,60	18,14	19	19,52	18,2	17
Total UFA		76,77	79,39	80,17	80	79,29	80,1	81,82
P/S index		1,04	1,56	1,91	1,74	1,48	1,97	2,16
UFA/SFA ratio		3,30	3,85	4,42	4,21	4,06	4,40	4,81

*P* poly-insaturated, *S* saturated, *SFA* Saturated fatty acids, *UFA* Unsaturated fatty acids

P/S index is known to be associated with healthy impact of oil diet, being in the range of 1–2,2 for our analyzed and cited Argan oils makes them suitable edible oils for human consumption, while the one of olive oil is around 0,5 [14].

**Fig. 3:**
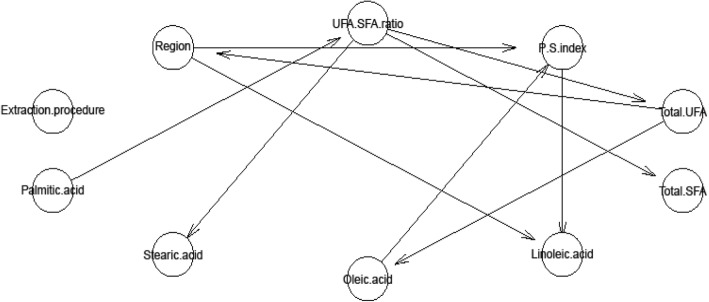
Directed acyclic graph representing possible region and procedure extraction connexions with fatty acids composition

To explain probabilistic interactions connecting variables and particularly, the impact of cultivation region of Argan tree, Bayesian networks modelling were used. We considered 10 nodes as represented in Fig. 3. Pearson’s correlation coefficients among chemical traits in Argan oil are presented and region cultivation and extraction procedure in Table 5. No significant correlation (*P* > 0.05) was found concerning relationship between extraction procedure and fatty acids composition (Table 5; Fig. 3). However, fatty acids composition was influenced by two parameters (region of cultivation and P/S index). In this study, “P/S index” has a double connection: “P/S index” is negatively related with oleic mono-unsaturated acid “Oleic acid” (*r* = − 0.946, *p* = 0.001) and on the other hand, positively correlated with linoleic poly-unsaturated acid “Linoleic acid” (*r* = 0.987, *p* = 0.002).

**Table 5:** Pearson’s correlation coefficients between fatty acids composition, region and the extraction procedure

		Region	Extraction procedure	Palmitic acid	Stearic acid	Oleic acid	Linoleic acid	Total SFA	Total UFA	P/S index	UFA/SFA ratio
Region	r	1									
	*P*-value	–									
Extraction procedure	r	0,059	1								
	*P*-value	0,900									
Palmitic acid	r	−0,861*	−0,035	1							
	*P*-value	0,028	0,947								
Stearic acid	r	−0,817*	−0,062	0,990**	1						
	*P*-value	0,025	0,895	0,000							
Oleic acid	r	−0,569	−0,391	0,944**	0,843*	1					
	*P*-value	0,182	0,386	0,005	0,017						
Linoleic acid	r	0,899*	0,510	−0,966**	−0,966**	−0,996**	1				
	*P*-value	0,038	0,380	0,008	0,008	0,000					
Total SFA	r	−0,785*	−0,054	0,999**	0,996**	0,863*	−0,968**	1			
	P-value	0,036	0,909	0,000	0,000	0,012	0,007				
Total UFA	r	0,748	0,112	−0,961**	−0,965**	−0,861*	0,981**	−0,964**	1		
	*P*-value	0,053	0,811	0,002	0,000	0,013	0,003	0,000			
P/S index	r	0,699	0,157	−0,992**	−0,961**	−0,946**	0,987**	−0,969**	0,961**	1	
	*P*-value	0,081	0,737	0,000	0,001	0,001	0,002	0,000	0,001		
UFA/SFA ratio	r	0,764*	0,006	−0,991**	−0,992**	−0,858*	0,963**	−0,993**	0,972**	0,976**	1
	*P*-value	0,046	0,989	0,000	0,000	0,014	0,009	0,000	0,000	0,000	–

*r* Pearson’s correlation coefficients

*P < 0.05; **P < 0.01

Additionally, Fig. 3 shows that “Linoleic acid” was positively influenced by the geographical localization “Region” (*r* = 0.899, *p* = 0.038).

## Discussion

### Argan oil yield

Yields of Argan fruit and oil depend on cultural techniques, environmental conditions, cultivars and extraction procedure [18]. Extraction by solvent gives generally the best oil yields and mechanization of the whole process of extraction is being more and more improved for industrial and marketing needs.

### Physicochemical properties of Argan oil

Practically, physicochemical parameters of Argan oil give indication about the technological quality of the oil and its end use, as well as its destination for cosmetic or diet purposes. Tunisian Argan oil displayed valuable qualitative parameters proving its competitiveness among Moroccan and Algerian oils. Our study showed that Tunisian Argan oil could be considered as extra virgin edible oil as well as for cosmetic use.

### Unsaponifiable fraction of Argan oil

Our study showed that polyphenols contents of Argan oil differ according to country origin and extraction procedure. Bibliographic prospection showed that Argan oil polyphenols content ranged from 6 to 150 ppm approximately [19] and that the polyphenol content of virgin Argan oil is lower than that of virgin Olive oil but higher than that of other edible vegetable oils [17]. Owing to its valuable content in polyphenol, Tunisian Argan oil would harbor nutraceutical properties and exert health benefits mainly through its antioxidant capacity. Interestingly, polyphenols extracted from virgin Argan oil were shown to exert anti-proliferative and pro-apoptotic effects on human prostate cancer cell lines [16]. More specifically, Argan oil polyphenols were responsible for the insulin signaling cascade disruption, supporting their putative anti-diabetic promising action [20]. Another domain of application of the argan oil in relation with its richness in polyphenols is the dermocosmetology, for skin care and protection.

Being rich in carotenoids (1,8 ppm) in comparison with Moroccan and Algerian samples (below 1 ppm), Tunisian Argan oil could be therefore a valuable dietary source of vitamin A since β carotene is a vitamin A precursor [21]. Although there is no recommended dietary allowances for carotenoids in the United States nor in Europe [22], through their antioxidant activity, carotenoids have been recently recognized as efficient against serious pathologies such as cancer, pulmonary disorders, heart disease and degenerative eye disease, and as regulators of the immune response system [22, 23]. Biologically, both polyphenols and carotenoids possess radical scavenging and antioxidant activities that are responsible for their main biological functions, pharmacological properties and nutraceutical roles [24].

### Fatty acid composition

Physico-chemical properties and qualitative range of Argan oil would depend primarily on its fatty acid composition [25]. Fatty acids play an important role on human health promotion, and since they cannot be synthesized by the human body they must be supplied by diet [23].

Generally, fatty acid composition of Argan oil is mainly dominated by UFA (75–85%) (i.e. oleic acid followed by linoleic acid) followed by SFA (15–25%) (i.e. stearic acid followed by palmitic acid). Interestingly, oleic acid content of Tunisian Argan oil is somehow close to that of Olive oil.

#### Oleic acid

Oleic acid is a mono saturated fatty acid that provides the human body with numerous types of health benefits. It acts in a very polyvalent way through a wide panoply of functions by protecting cells from free radical damage via antioxidant activity, increasing fat burning to help with weight loss, preventing type 2 diabetes and reducing the insulin resistance, lowering blood pressure and aiding the circulatory system, preventing ulcerative stomach, reducing all symptoms commonly related to arthritis, and generating brain myelin. Additionally, oleic acid is known for moisturizing the skin, strengthening the hair, preventing wrinkles and its anti-aging properties.

Tunisian Argan oil, rich in oleic acid, would display modulatory effects on cancer, autoimmune and inflammatory diseases, besides its ability to facilitate wound healing. Thus, the administration of Argan oil containing diets may improve the immune response associated to a more successful elimination of pathogens such as bacteria and fungi [26].

#### Linoleic acid

Initially characterized as an anticancer component, linoleic acid has been also shown to prevent the development of atherosclerosis, reduce body fat while improving lean body mass, and modulate immune and/or inflammatory responses [27]. Linoleic acid is known to exert a wide range of physiological functions such as antidiabetic, antihypertensive, antiobese and anticarcinogenic properties, meaning that argan oil would be effective to prevent lifestyle diseases or metabolic syndromes [28]. Physiologically, it helps the body in vitamins A and D absorption, regulates growth of new cells.

#### Stearic acid

Compared with other cholesterol-raising SFAs, stearic acid lowers LDL cholesterol, was neutral with respect to HDL cholesterol, and directionally lowered the ratio of total to HDL cholesterol [29]. When compared with UFA, stearic acid tended to raise LDL cholesterol, lower HDL cholesterol, and increase the ratio of total to HDL cholesterol. Thus, stearic acid would be a good substitute for cholesterol-raising SFA (lauric, myristic, and palmitic) for solid fat applications [30].

#### Palmitic acid

Even being known as undesirable at high amount because of its negative impact on health, particularly for being involved in cardiovascular diseases, recently, palmitic acid, in moderation, have been shown to display mild antioxidant and anti-atherosclerotic properties.

Palmitic acid has been thought for many years to raise cholesterol levels, however, when combined with linoleic acid, it does not raise cholesterol. Cosmetic use of these fatty acids has been reported by the US Food and Drug Administration in 2016, including several product categories like skin care, makeup, shaving preparation, soaps and lotions, nail and hair products [31].

#### P/S index

The content of fatty acids as well as the ratio between unsaturated and saturated fatty acids are important parameters in the determination of nutritional value of oil. Oils and fats with higher value of P/S index than 1, like all analyzed Argan oils, are considered to have nutritional value. Several studies indicated that a high value of P/S index means less accumulation of lipids in the body.

The 2015–2020 Dietary Guidelines for Americans Committee (DGAC) gave more emphasis in optimizing fat quality than reducing total fat intake. Given that high SFA content in foods can have unfavorable health effects if consumed at excessive dietary levels, the DGAC recommends lower intake of dietary saturated fats, and that sources of saturated fat should be replaced with unsaturated fat, particularly polyunsaturated fatty acids [32]. One example of improving nutritional food profile would be to achieve a better polyunsaturated fat to saturated fat ratio [30]. The calculated P/S index and UFA/SFA ratio of Argan oils revealed equilibrated values, concordantly with the DGAC recommendations.

## Conclusions

Our comparative analysis between several Argan oils showed that their physicochemical properties and chemical composition depend on the origin and the extraction procedure. These variabilities in Argan oil composition and physicochemical properties could arise from genetic diversity between cultivars, pedo-climatic conditions of growth and extraction procedure. However, Tunisian Argan oil displayed globally better nutritional and qualitative characteristics making it classified as extra virgin oil. Indeed, although the physico-chemical properties and the chemical composition of Argan oil depend on several factors such as the cultivar, the fruit ripeness, the pedo-climatic conditions of culture, and the extraction procedure, Tunisian Argan oil still constitute a promising and competitive alternative for nutraceutical and cosmeceutical uses. Taken together, all these data and results related to the physicochemical properties and chemical composition of Argan oil, particularly Tunisian one, are likely to be responsible for its nutraceutical properties and beneficial and healthful effects.

## Abbreviations

DGAC: Dietary guidelines for Americans Committee
FAME: Fatty acid methyl esters
GC/MS: Gas chromatography/mass spectrophotometry
HDL: High density liporotein
LDL: Low density lipoprotein
SFA: Saturated fatty acids
UFA: Unsaturated fatty acids
